# Long-term prognostic value of myocardial salvage assessed by cardiovascular magnetic resonance in acute reperfused myocardial infarction

**DOI:** 10.1186/1532-429X-13-S1-P181

**Published:** 2011-02-02

**Authors:** Ingo Eitel, Steffen Desch, Suzanne de Waha, Georg Fuernau, Matthias Gutberlet, Gerhard Schuler, Holger Thiele

**Affiliations:** 1 Heart Center Leipzig, Leipzig, Germany

## Introduction

In acute myocardial infarction, cardiovascular magnetic resonance (CMR) can retrospectively detect the myocardium at risk and the irreversible injury. This allows for quantifying the extent of salvaged myocardium after reperfusion as a potential strong end point for clinical trials and outcome. We have previously shown that the myocardial salvage index (MSI) assessed by CMR is a strong indicator of 6-months clinical outcome after ST-segment elevation myocardial infarction (STEMI).

## Purpose

Aim of this study was to investigate whether the early benefits of such findings are sustained at long-term clinical follow-up in STEMI patients undergoing primary angioplasty.

## Methods

We analyzed 208 consecutive STEMI patients undergoing primary angioplasty <12 h after symptom onset. T2-weighted and contrast-enhanced CMR was used to calculate the myocardial salvage index (MSI). Patients were categorized into 2 groups defined by the median MSI. The primary end point of the study was occurrence of major adverse cardiovascular events (MACE) defined as death, reinfarction, and new congestive heart failure at long-term clinical follow-up.

## Results

The median MSI was 48 (interquartile range 27 to 73). Long-term follow-up was available in 199 patients (96%) at a median of 17.4 months (interquartile range 10.9 to 20.3). MACEs occurred in 29 patients (15%), with a significantly lower event rate in the MSI ≥ median group (5 versus 24 events, p<0.001). MSI ≥ median was a significant predictor for a favourable long-term clinical outcome on multivariable Cox regression analysis after adjustment for established prognostic markers (Hazard ratio: 0.23, confidence interval: 0.09 to 0.57, p<0.001).

## Conclusions

This study for the first time demonstrates that the MSI assessed by CMR predicts long-term clinical outcome in acute reperfused STEMI. Therefore, MSI assessment has important implications for patient prognosis as well as for the design of future trials intended to test new reperfusion therapy efficacy.

